# Prehospital Use of the Intubating Laryngeal Mask Airway in Patients with Severe Polytrauma: A Case Series

**DOI:** 10.1155/2009/938531

**Published:** 2009-06-25

**Authors:** Andrew M. Mason

**Affiliations:** Suffolk Accident Rescue Service, Turret House, 2 Turret Lane, Ipswich IP4 1DL, UK

## Abstract

A case series of five patients is described demonstrating the utility of the intubating laryngeal mask airway in the prehospital setting, both as a primary airway rescue device and as a bridge to tracheal intubation. All patients were hypoxaemic, had sustained severe polytrauma and were trapped in their vehicles following road traffic collisions. A probability of survival study showed better-than-predicted outcomes for the group as a whole.

## 1. Case Studies

The study group consisted of consecutive trapped patients with severe polytrauma who presented over a period of nineteen months. The principle reason for use of the intubating laryngeal mask airway (iLMA or LMA Fastrach - LMA North America Inc., San Diego, Calif, USA) in these cases was an inability to establish or maintain adequate oxygenation (defined as a pulse oximetry reading of at least 90%) using basic airway techniques such as opening the airway, insertion of an oropharyngeal or nasopharyngeal airway (or both) and the administration of high-flow supplemental oxygen therapy via a nonrebreathing mask or a bag-valve-mask ventilation device. In some cases, this hypoxaemia was accompanied by an inability to prevent aspiration of blood by regular suctioning of the airway. All patients were continuously monitored by pulse oximetry and capnography using a Capnocheck II device (Smiths Industries Medical Systems, Wis, USA). Where patients were intubated via the iLMA, a flexible, reinforced tracheal tube with an atraumatic tip designed specifically for use with the iLMA (Euromedical Industries, Kedah, Malaysia) was employed on each occasion.

### 1.1. Case A

This 58-year-old male was the driver of a car which collided with a truck. He sustained serious head and facial injuries and was trapped in a sitting position for 60 minutes. The first paramedics at the scene found that he responded to painful stimuli but not to verbal commands with a Glasgow Coma Score (GCS) of 8 (eyes 1, motor 5, verbal 2). He was breathing spontaneously and his SpO2 was initially maintained around 95% by means of high-flow oxygen administered via a nonrebreathing mask (NRBM). However, it proved difficult to prevent aspiration of blood from his facial injuries despite frequent suctioning of the airway, and the patient became combative as his SpO2 level dropped to 80%. While still trapped in a sitting position, the patient was sedated with midazolam (5 mg IV bolus) and an iLMA was inserted without difficulty. Intermittent positive pressure ventilation (IPPV) was applied by means of a bag-valve device fitted with an oxygen reservoir, and the patient's SpO2 rose steadily to 100%. Because the SpO2 was optimal and the cuff of the iLMA was judged to be preventing further aspiration of blood, no attempt was made to intubate the patient via the iLMA either at the scene or on the journey to hospital. After arrival at hospital, the author intubated the patient via the iLMA at the first attempt. A CT scan revealed severe diffuse brain injuries and major facial damage including a Le Fort II fracture of the maxilla. The patient spent a total of eight weeks on the neurosurgical intensive therapy unit but went on to make an excellent recovery with no cognitive impairment.

### 1.2. Case B

**Figure 1:**
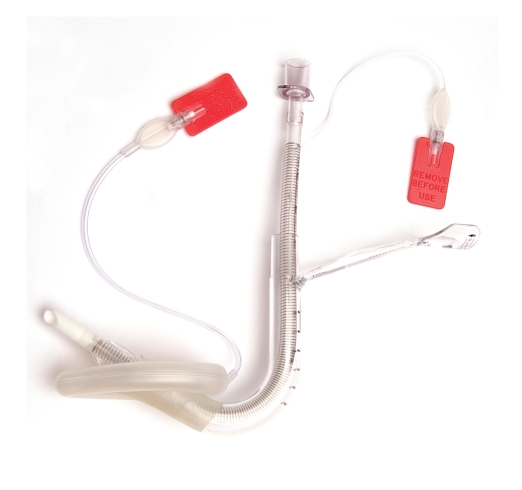
The single-use LMA Fastrach (LMA North America Inc., San Diego, Calif, USA) with its dedicated single-use reinforced tracheal tube (image courtesy of The Laryngeal Mask Company Limited, Jersey, Channel Islands).

This 48-year-old female was a front passenger in a van involved in a head-on collision with a car and was subsequently trapped in a sitting position for 75 minutes. On initial assessment she was able to respond to verbal commands (GCS 14; eyes 3, motor 6, verbal 5), and there was brisk bleeding from the nose and mouth as a result of extensive facial injuries. This bleeding was unusually persistent and was only later shown to be secondary to a low platelet count (69 × 10^9^/L) due to idiopathic thrombocytopenic purpura. Other injuries included an open fracture of the left wrist and a fracture dislocation of the left elbow. She was given high-flow oxygen via an NRBM which initially maintained her SpO2 around 98%. However, bleeding into the airway became an increasing problem despite frequent suctioning, and her SpO2 was seen to fall to 88%. Extrication and transport in a supine position were considered to carry a major risk of aspiration if undertaken with an uncontrolled airway. Consequently, an iLMA was inserted after sedation with midazolam (two 5 mg IV boluses) with the patient still trapped in a sitting position (Figure 1). Spontaneous respiration was augmented with a bag-valve-mask device (BVMD) fitted with an oxygen reservoir and the SpO2 rose rapidly to 100%. In view of the good response, it was judged unnecessary to intubate the patient via the iLMA either at the scene or en route to the nearby hospital. On arrival at hospital the duty anaesthesiologist requested removal of the iLMA in order that RSI could be performed under direct laryngoscopy. Despite the use of a gum elastic bougie, multiple intubation attempts by two experienced anaesthesiologists failed due to an anatomical anomaly (recession of the mandible) and the continuing bleeding into the oropharynx. The iLMA was therefore reinserted and, following a period of reoxygenation, the author intubated the patient via the iLMA at the first attempt. The patient went on to make a full recovery.

### 1.3. Case C

This 59-year-old female was the driver of a car which was involved in a head-on collision with another vehicle and was trapped in a sitting position for 45 minutes. She was unresponsive to verbal or painful stimuli with a GCS of 3. At the scene she was hypotensive, tachycardic and tachypnoeic with an SpO2 of 82% despite assisted ventilation using a BVMD. Consequently, she was given midazolam (5 mg IV bolus) to relax the jaw and an iLMA was inserted while the patient was still trapped in a sitting position. IPPV via the iLMA brought about a rise in SpO2 to 100% and assisted ventilation was continued throughout the journey to hospital. On arrival at the hospital the author intubated the patient via the iLMA at the first attempt under neuromuscular blockade. Her injuries included major head trauma consisting of a compound fracture of the base of the skull, brainstem haemorrhage and major global brain contusions, a left-sided flail chest, lung contusions and (later in hospital) a tension pneumothorax, together with serious injuries to her lower limbs including bilateral mid-shaft femoral fractures, a left inter-trochanteric femoral fracture and a fracture of the right femoral neck. The patient's condition remained critical for the first week and then showed slow but steady improvement. She was eventually discharged home almost a year later with a residual left hemiparesis and some mild-to-moderate cognitive impairment.

### 1.4. Case D

This 43-year-old male was the driver of a car which left the road in icy conditions and collided with a tree. He was trapped in his vehicle for a total of 20 minutes, and a passing off-duty paramedic found that he was unresponsive to all stimuli with a GCS of 3. He also noted that the patient's pupils were fixed and dilated and respirations were agonal at a rate of only six breaths-per-minute. The paramedic began IPPV with a BVMD and a rapid extrication procedure was undertaken as soon as help arrived. An iLMA was then inserted at the roadside without the need for drugs to facilitate insertion. There was no palpable radial or carotid pulse and the application of cardiac monitoring electrodes revealed the presence of pulseless electrical activity (PEA), so CPR was commenced and continued throughout the journey to hospital. No SpO2 reading could be obtained in the prehospital phase of treatment. At the hospital the duty anaesthesiologist requested the removal of the iLMA and a tracheal tube was then placed under direct laryngoscopy without the need for RSI drugs. Despite all measures, the PEA rhythm degraded to asystole and this failed to respond to standard treatment. Resuscitation was therefore abandoned. A post-mortem examination revealed a fracture dislocation at the level of the 2nd cervical vertebra with partial transection of the spinal cord at this level, multiple rib fractures with bilateral lung contusions and a right haemothorax, a fracture of the pelvis and multiple lacerations to both the liver and spleen.

### 1.5. Case E

This 19-year-old male was a front passenger in a car which was involved in a high-speed head-on collision with a truck. There was massive frontal damage to the car and the patient was heavily trapped in the wreckage for a total of 90 minutes. Both driver and passenger sustained critical injuries and extrication of the driver was undertaken first. Throughout this time the passenger was totally unresponsive with a GCS of 3. The very limited access to the passenger meant that the only treatment that could be given was IPPV with a BVMD to support spontaneous respiration. As soon as access to the passenger was obtained, his respiratory rate was found to be 33 breaths-per-minute. Radial pulses were impalpable but the carotid pulse was 160 beats-per-minute (rate confirmed by cardiac monitor). His SpO2 was 77%, so midazolam (2.5 mg IV bolus) was given to relax the jaw and an iLMA was inserted at the roadside. Spontaneous respiration was then augmented with a BVMD, but the SpO2 reading could not be elevated above 91%. Chest expansion was noted to be equal on both sides and there was normal air entry to all lung fields. Despite the insertion of a tracheal tube via the iLMA en route to hospital and continued IPPV, the patient's SpO2 remained at 91%. The low oxygen saturation was subsequently shown to be due to severe bilateral pulmonary contusions. Other injuries included bilateral closed fractures of the femoral shafts, bilateral open tibial fractures, an open fracture of the left radius and a closed fracture of the right radius. A CT brain scan revealed only minimal cerebral oedema and the patient went on to make a full recovery from his injuries.

## 2. Results

Key clinical findings for each patient are shown in Table 1.

Copies of the full clinical records of each patient were anonymised and then sent to the Trauma Audit and Research Network (TARN) in the UK for independent analysis, and an injury severity score (ISS) [1] and probability of survival (Ps) [2] figure were provided for each patient.

The systolic blood pressure (BP) was estimated at the scene initially according to the presence (systolic BP >80 mmHg) or absence (systolic BP <80 mmHg) of a radial pulse. Formal measurement of BP was not undertaken in the early stages of rescue because of attention to tasks with a higher priority.

The mean probability of survival for the group as a whole was 37.4%, with an actual survival rate of 80%.

In four out of the five cases (A, B, C, E), intravenous midazolam was administered (A: 5 mg, B: 10 mg, C: 5 mg, E: 2.5 mg) as a single agent to facilitate insertion of the iLMA. The only patient in whom use of midazolam was not necessary (D) was the sole nonsurvivor.

## 3. Discussion

The iLMA is designed to permit blind intubation of the trachea with a dedicated wire-reinforced tracheal tube (Figure 1). However, it also functions as a rescue airway device in its own right. Although the iLMA has been included in guidelines for the management of the difficult airway by the American Society of Anesthesiologists [3] and is used widely within hospitals, there are a limited number of reports of its use in the prehospital environment [4–7], despite a range of features that make it highly suitable for this setting (Table 2).

**Figure 2:**
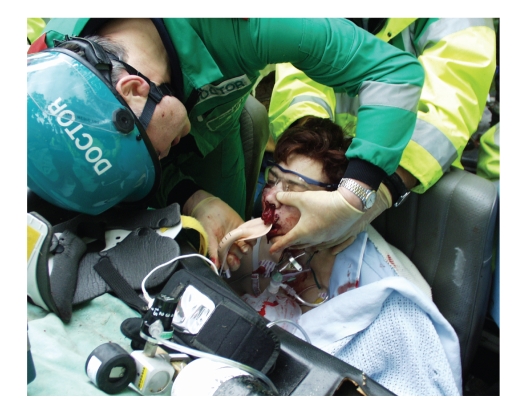
Insertion of the iLMA with manual in-line axial stabilisation of the cervical spine applied by an assistant (image shown with the full informed consent of the patient (Case B)).

The mechanism of injury in each case raised the possibility of significant trauma to the cervical spine—something that could not be excluded at the scene due to either impaired consciousness (A, C, D, E) or the presence of other distracting injuries (B). The iLMA was selected to establish the airway because manipulation of the head and neck is not necessary for its insertion, and because of the speed with which it can be deployed in situations where there is limited access to trapped patients. All the patients had the iLMA inserted with their head and neck maintained in neutral alignment by an assistant as advised by the manufacturer (Figure 2). 

Concern is sometimes expressed about the relative lack of protection that laryngeal mask devices afford against aspiration of regurgitated gastric contents in nonfasting subjects. However, in a multicentre hospital-based CPR study [8], the incidence of aspiration with the LMA was less than 1%—much less than has been shown to occur with the unprotected airway when other ventilatory techniques such as bag-valve-mask ventilation are used during resuscitation [9]. In trauma, and particularly in those with maxillofacial injuries, the risk of aspiration of gastric contents is, in any case, less than the risk of aspiration of blood [10], and the cuff of the LMA has been shown to afford effective protection against the aspiration of blood or dye within the oropharynx [11, 12]. By extrapolation, it seems reasonable to conclude that the cuff of the iLMA will provide similar protection.

Cervical spine injuries occur in 2–5% of blunt trauma patients but, provided that manual in-line neck stabilisation is applied, evidence suggests that rapid sequence induction of anaesthesia followed by direct laryngoscopy and oral intubation (RSI) is a safe procedure in patients with injuries to the cervical spine [13]. However, safety studies have only looked at intubations undertaken by specialists working within the hospital environment. The safety of intubation under direct laryngoscopy for those with spinal injuries cannot be taken for granted in the chaotic prehospital environment, particularly when operators have limited clinical experience, inadequate assistance, are denied the use of RSI drugs, and where there is restricted access to trapped patients. In these circumstances, alternative strategies need to be considered for establishing an airway. Because the iLMA must be inserted with the patient's head and neck maintained in neutral alignment, the device could be particularly suitable for use in trauma where injury to the cervical spine has not been excluded.

Both manikin and human studies have shown that iLMA insertion and subsequent ventilation using the device can be achieved readily by nonphysicians as well as by those with minimal training [14–18], suggesting that it could be a suitable airway for use by paramedics. Early studies suggested that a learning curve of approximately twenty cases existed for proficiency in tracheal intubation via the iLMA [19]. However, subsequent refinements to the recommended insertion technique have managed to improve first-time intubation success rates significantly [20]. The recommended technique for intubation via the iLMA is sometimes called the “Chandy” manoeuvre, named after the UK anaesthesiologist, Dr. Chandy Verghese. The first part of the Chandy manoeuvre involves grasping the iLMA by its handle and moving it back and forth in the sagittal plane while noting the rise-and-fall of the chest (tidal volume) together with the resistance to manual ventilation. This optimises ventilation through the device which occurs when the distal airway aperture in bowl of the mask is directly opposite the laryngeal inlet. The second part of the manoeuvre involves lifting the handle of the iLMA at 45% to the horizontal plane of the patient's chest. This helps to align the angled ramp at the distal end of the airway aperture with the longitudinal axis of the upper trachea, so facilitating direct and unhindered passage of the tip of the tube into the upper trachea. Use of the Chandy manoeuvre can be expected to improve first-time intubation rates with the iLMA to levels approaching 100%.

In view of the advantages listed in Table 2, it seems reasonable to propose that the iLMA could be deployed as a primary airway rescue device by most grades of emergency personnel, allowing rapid correction of critical hypoxaemia followed, if required, by unhurried attempts at intubation with minimal interruption to oxygenation. Where necessary, iLMA-guided tracheal intubation could be postponed until someone with the necessary expertise is available.

It is worth noting that, of the four patients in this study who were intubated via the iLMA (A, B, C, E), only one (E) had the procedure performed in the prehospital phase of treatment, and this resulted in no enhancement of oxygenation over that obtained using the iLMA alone. Therefore, if the better-than-predicted outcomes for this group of patients were the direct consequence of the airway management that they received, it seems likely that this was due to the prompt correction of hypoxaemia/hypercarbia by the iLMA and its ability to prevent aspiration of blood, rather than due to the subsequent intubation process itself. If this is a general principle for prehospital trauma care, then the attempted intubation of patients by paramedics using direct laryngoscopy—with all its attendant problems of prolonged on-scene times [21], increased mortality with poorer neurological outcomes for patients with traumatic brain injury [22, 23], and unrecognised tube malplacement [24]—may be an unnecessary risk for most patients. Exceptions would include patients with airway burns, for example, who are unsuitable candidates for supraglottic airway device placement. If rapid correction of hypoxaemia is more important than the placement of a tracheal tube for the majority of patients, then it follows that other supraglottic airway devices may be equally as valuable as the iLMA in the prehospital phase of treatment.

Use of sedative or anaesthetic drugs by paramedics to secure the airway in the prehospital environment is a controversial topic [25]. Very few paramedics in the UK are permitted to administer neuromuscular blocking agents (NMBAs) and standard operating procedures often restrict the use of parenteral benzodiazepines to the control of persistent seizures [26]. Midazolam, a short-acting benzodiazepine, is the author's preferred drug for facilitating insertion of LMA devices in the prehospital setting, and it has been shown that low-dose midazolam (up to 5 mg) is both safe and effective when used by paramedics for prehospital RSI, with no adverse cardiovascular effects when compared with etomidate [27]. It is worth noting that all four patients who received midazolam in this study went on to survive, and the one patient who accepted the iLMA without sedation was the sole nonsurvivor. It has been shown that there is no difference in the success rate for intubation via the iLMA between chemically paralysed and nonparalysed patients [28]. However, the incidence of reflex coughing is likely to be higher in nonparalysed individuals, and the possible implications of coughing in the presence of brain injury and raised intracranial pressure need to be borne in mind. 

The small number of patients included in this study makes it impossible to draw any firm conclusions concerning the value of the iLMA for prehospital trauma care, since the apparent benefit in terms of survival could have arisen by chance alone. However, the rapid reversal of hypoxaemia in four of the five cases coupled with the prevention of ongoing aspiration of blood is encouraging and would appear to justify further trials of the iLMA as an airway adjunct for use by paramedics in the prehospital setting.

Other supraglottic airway devices (SADs) that could be suitable for use when there is restricted access to trapped casualties include the oesophageal-tracheal Combitube (Tyco-Kendall, Mansfield, Mass, USA), the LMA Proseal with its optional insertion handle, LMA CTrach and the LMA Supreme (LMA North America Inc., San Diego, Calif, USA), and the King Laryngeal Tube (LT) family of devices (King Systems Corp, Noblesville, Ind, USA). Of all the SADs currently available, only the iLMA (LMA Fastrach) and the LMA CTrach permit seamless progression to tracheal intubation with no interruption in ventilation and oxygenation. However, users of the iLMA should be aware that blind intubation with the device is contraindicated in the presence of known pharyngeal pathology or where the patient has sustained trauma to the larynx. 

It should also be appreciated that SADs do not provide a definitive airway because they do not seal within the trachea like a cuffed tracheal tube. Other drawbacks of SADs include the possibility of periglottic trauma and laryngospasm. Users should also be aware that epiglottic downfolding during insertion of the iLMA (and other SADs) can restrict the ability to ventilate the patient. In the event of epiglottic downfolding with the iLMA, emergency providers need to be aware of techniques such as the “up-down” manoeuvre to overcome this problem.

## 4. Conclusions

This study shows the potential value of the iLMA as a device for rapid control of the airway in the prehospital setting, particularly when there is restricted access to trapped casualties. It also demonstrates that midazolam can be a useful single agent in low dosage to facilitate insertion of an iLMA into patients with severe polytrauma. Further work is needed to determine the suitability of the iLMA as an airway management device for use by paramedics.

**Background**

The author is a member of a group of volunteer physicians providing on scene support for the statutory ambulance service in a largely rural area of eastern England. The physicians respond to the scene in their own marked vehicles and carry equipment and drugs not found on front line ambulances in the region. Callouts come directly from the ambulance control centre and may be simultaneous with the mobilisation of an ambulance, or at the request of emergency personnel already at the scene. The responding physicians come from a number of different medical specialities, including Anaesthesiology, Accident and Emergency Medicine and Family Medicine. Some physicians are highly experienced in rapid sequence induction of anaesthesia and intubation (RSI) techniques, whereas others confine themselves mainly to placement of supraglottic airway devices, with or without the administration of sedative agents. Robust clinical governance standards and an ongoing training programme ensure that the methods of advanced airway management employed by each physician match both the individual's training and experience. All patients in this study were treated by the author who is able to undertake RSI, if necessary.

## Figures and Tables

**Table 1:** Summary of clinical findings.

					Pre-iLMA insertion									
Case	Gender	Age (yrs)	Time trapped (+journey time to hospital) (mins)	Sedation given (IV Midazolam)	GCS	RR (b/min)	Systolic BP (mmHg)	Pulse (b/min)	SpO2 % (pre- iLMA)	SpO2 % (post- iLMA)	iLMA used to facilitate tracheal intubation (TI)	ISS	Ps%	Survived to discharge from hospital
A	M	58	60 (+47)	Yes (5 mg bolus)	8/15 (Combative)	24	>80	68	80	100	Yes (in ED at hospital)	30	57	Yes
B	F	48	75 (+6)	Yes (5 mg bolus ×2)	14/15	25	>80	110	88 (off O_2_)	100	Yes (following failed RSI under direct laryngo-scopy in ED)	22	96	Yes
C	F	59	45 (+8)	Yes (5 mg bolus)	3/15	40	<80	118	82	100	Yes (in ED)	59	3	Yes
D	M	43	20 (+7)	No	3/15	6	<80	PEA	No reading	No reading	No (iLMA removed in ED then TI by direct laryngo-scopy)	57	6	No
E	M	19	90 (+21)	Yes (2.5 mg bolus)	3/15	33	<80	160 (cardiac monitor)	77	91 (post-iLMA and post-TI)	Yes (in ambulance)	34	25	Yes

**Table 2:** Advantages of the iLMA for emergency prehospital care.

1	Insertion and ventilation can be achieved easily by persons with minimal training [14–18].
2	Functions as a rescue airway device in its own right.
3	Laryngoscopy unnecessary.
4	Neutral alignment of head & neck a prerequisite for insertion.
5	Requires an interdental gap of only 20 mm.
6	Neuromuscular blockade not required for insertion.
7	Can be introduced blindly with one hand from any position.
8	No need to insert a finger into patient's mouth.
9	Rigid airway tube resists occlusion by biting.
10	Facilitates seamless progression to tracheal intubation.
11	Permits ventilation between/during intubation attempts.
12	Available as a disposable single-use device.
